# Accumulated Long-Term Exposure to Workplace Bullying Impairs Psychological Hardiness: A Five-Year Longitudinal Study among Nurses

**DOI:** 10.3390/ijerph17072587

**Published:** 2020-04-10

**Authors:** Kristina Vaktskjold Hamre, Ståle Valvatne Einarsen, Øystein Løvik Hoprekstad, Ståle Pallesen, Bjørn Bjorvatn, Siri Waage, Bente E. Moen, Anette Harris

**Affiliations:** 1 Department of Psychosocial Science, University of Bergen, 5015 Bergen, Norway; ruw005@uib.no (K.V.H.); Stale.Einarsen@uib.no (S.V.E.); Oystein.Hoprekstad@uib.no (Ø.L.H.); Staale.Pallesen@uib.no (S.P.); 2 Norwegian Competence Center for Sleep Disorders, Haukeland University Hospital, 5021 Bergen, Norway; Bjorn.Bjorvatn@uib.no (B.B.); siri.waage@uib.no (S.W.); 3 Department of Global Public Health and Primary Care, University of Bergen, 5018 Bergen, Norway; Bente.Moen@uib.no

**Keywords:** bullying, psychological hardiness, personality, stress, coping, nurses

## Abstract

Personality has been hypothesized to act as antecedent as well as an outcome of workplace bullying. Still, investigations on the longitudinal relationship between bullying and personality are scarce. We investigated the relationship between accumulated exposure to bullying at work and subsequent changes in psychological hardiness. Additionally, we examined whether hardiness predicted subsequent exposure to bullying. The data were based on the Survey of Shiftwork, Sleep, and Health (SUSSH), a cohort study with annual surveys among Norwegian nurses. The participants who completed standardized instruments measuring exposure to bullying behavior at T1 (2008/09) to T4 (2012) and psychological hardiness at T1 (2008/09) and T5 (2012) were included (*n* = 938). The results showed that accumulated exposure to bullying (sum of exposure from T1–T4) was associated with reduced psychological hardiness at T5, adjusted for age, sex, and hardiness at baseline (*β* = –0.16, t = –5.70, *p* < 0.001). Accumulated exposure to bullying behaviors explained 2.3% of the change in hardiness. Less hardy individuals experienced higher levels of subsequent exposure to bullying behaviors, adjusted for age, sex, and bullying at baseline (*β* = –0.04, *t =* –2.21 *p* < 0.05). Long-term accumulated exposure to bullying behaviors seemed to be a stronger predictor for changes in hardiness as compared to hardiness in predicting exposure to bullying.

## 1. Introduction

Workplace bullying represents a major hazard to the health and well-being of those exposed, being a serious stressor that is associated with a wide range of mental health problems [1,2,3], as well as with an absence from work [4]. Among nurses, bullying has been associated with increased psychological distress [5], low concentration, and productivity [6]. It is claimed that bullying triggers greater strain than do other work-related stressors combined [7]. Workplace bullying might take different shapes and forms, including verbal hostility, ridicule, assignment to unfair or dangerous work tasks, or plain acts of social exclusion [8]. Bullying concerns the accumulated and on-going exposure to such treatment. Hence, workplace bullying can be formally defined as a situation where one person, over a period of time, perceives him/herself to be at the receiving end of on-going negative actions from one or other organization members, finding it difficult to defend him/herself against these actions [8]. The definition implies three main characteristics: (1) an employee becomes the target of systematic negative and unwanted social behavior, (2) the exposure occurs over a long period, and (3) the target cannot easily escape the situation nor stop the negative treatment [9].

Systematic and long-term exposure to workplace bullying will normally trigger strong stress reactions [10], even to the degree of producing posttraumatic stress symptoms [11], with accompanying changes in basic psychological functioning, like avoidance behavior, irritability, and anger [12]. From the early works of Leymann [13], it has been claimed that the interpersonal trauma that is caused by bullying might trigger changes in both personality and in one’s basic coping strategies and abilities, being traumatic to the extent that it might shatter an individual’s basic assumptions regarding him/herself and the world [14]. Hence, long term exposure might change individual dispositions [15,16] and threaten basic physiological needs, such as the need for relatedness, need for competence, and need for autonomy [17]. This long-term blocking of one’s basic psychological needs might in itself cause changes in one’s individual dispositions [18], potentially decreasing one’s coping resources and increase one’s vulnerability when facing new stressors (see also [19]). Still, there is not much empirical evidence on the relationship between long-term exposure to bullying and changes in individual disposition and coping mechanisms, thus reducing our ability to identify relevant antecedents and consequences that are involved in the complexity of workplace bullying [20]. Therefore, the main aim of the present study was to investigate the relationship between accumulated and long-term exposure to bullying behaviors at work and subsequent changes in psychological hardiness, an individual disposition that is regarded as a personal resource that might help individuals to cope with stressful situations. 

The focus on individual factors is one of two main perspectives, normally studied when risk factors of workplace bullying are examined [9]. The other perspective emphasizes how the immediate work environment and work design can trigger bullying processes [13]. At least four theoretical mechanisms explaining the relationship between individual dispositions and exposure to workplace bullying have been suggested in terms of the individual perspective [15]. First, in *the no relationship mechanism*, it is proposed that bullying is not associated with individual dispositions at all, serving as a null hypothesis. In his pioneering work on bullying in Sweden, Leymann [13] strongly supported such a position, arguing that bullying was merely down to problematic working conditions and flaws in leadership. The second mechanism, *the target-behavior mechanism*, suggests that specific individual dispositions may elicit aggressive behavior directed towards the focal person. The concept of the provocative target [21] and the victim precipitation theory [22] holds such an assumption, in that the target is assumed to demonstrate behaviors and reactions that may provoke others. The third mechanism, the *negative perceptions mechanism*, builds on the assumption that employees with a certain individual disposition have a higher risk than others of labelling negative events at the workplace as bullying and a lower threshold for perceiving others as hostile. The fourth mechanism, *the reversed causal mechanism*, suggests that individual dispositions should be considered to be a potential outcome of workplace bullying, rather than an antecedent. Leymann [13] argued strongly that any individual characteristic in terms of personality associated with exposure to bullying should be regarded as an outcome of the traumatic experience of bullying.

As the causal associations between individual dispositions and bullying may be multifold, longitudinal studies are needed in order to establish whether specific dispositions are risk factors for exposure, or whether bullying leads to changes in dispositions among those severely exposed [9]. Theoretically, personality factors are considered to be relatively stable dispositions, and should therefore not, in principle, be affected by social stress. However, traumatic events may be life-changing experiences [23,24], and sustained changes in mental health functioning among targets of bullying have been documented as late as five to seven years after the event [25]. Furthermore, research has shown that personality traits are malleable and susceptible to environment influence, even after the age of thirty [26], and both social relationships and work experiences can have a significant impact on personality [27]. In studies examining individual dispositions and distress at work, the former are often regarded as moderators of the relationship between stress exposure and employee outcomes. However, long-term exposure to severe stressors at work might also feed back to and produce changes in individual dispositions. How we perceive, cope with, and handle prolonged social stress is an individual factor that might be particularly vulnerable to change when experiencing severe distress. The developments of learned helplessness and hopelessness are well known psychological mechanisms in this regard [28]. Thus, bullying might not only be detrimental for the workers’ health and well-being, but also reduce employees’ coping resources, leaving them more vulnerable to a multitude of stressors at work.

Psychological hardiness is an individual disposition, which is usually regarded as a protective factor affecting how individuals cope and persevere when facing peril and severe demands, e.g., at work [29]. Psychological hardiness consists of three interrelated dispositions: involvement, control, and challenge [30]. A person’s tendency to participate actively in life and to have a real interest in the outside world is called involvement. Control is to believe and act on the basis that one can, through one’s own efforts, influence one’s circumstances. Challenge is the idea that change is a normal part of life, as opposed to a threat. Theoretically, the combination of these dispositions provides the person with the necessary courage and motivation needed in order to turn stressful experiences into opportunities for growth, instead of experiencing threat and lack of safety [31]. This can be related to transformative coping, where individuals meet stressors with an expanded perspective, a deeper understanding, and a positive expectation for handling the stressor [32]. This theoretical proposition is consistent with the principles of cognitive activation theory of stress (CATS) [33]. CATS postulates that an individual’s expectation of the outcome of a given situation is crucial to the physiological stress response and whether the stressor will cause health problems [33]. If the person has a negative (or no) response outcome expectancy when facing a stressor, like, for instance, bullying, the load might result in sustained activation manifested as a range of negative stress symptoms. Conversely, if the person has a positive coping expectation when facing stressors, the load will result in personal growth that makes the individual more robust and stronger in the face of similar situations in the future. 

The successful handling of previous stressful events has been attributed to the development of psychological hardiness [34], and research has shown that people high in psychological hardiness master stressful situations better than people who score low on this personality trait [35,36]. In a recent study of workers who were exposed to low levels of bullying behaviors at the workplace, hardiness was a protective factor in the bullying-anxiety relationship, yet did not moderate the bullying-depression relationship [37]. However, a range of studies have lately shown that individual dispositions that are similar to hardiness, such as sense of coherence [38], the ability to defend [39], internal locus of control [40], and problem-focused strategies [41], do not protect targets when facing bullying the way they are supposed to do theoretically [9]. In summary, these studies show that individual resources, such as hardiness, only have a protective effect in cases of low exposure to bullying behaviors at the workplace. Under severe exposure to bullying behaviors, the outcome might be relatively more detrimental for those who initially held high coping resources—suggesting that high intensity bullying is detrimental for all those exposed, irrespective of individual resources. 

Based on established stress theories such as CATS, it is normally expected that psychological hardiness will protect victims from the negative effect related to the stress released from, for example, exposure to workplace bullying. Nevertheless, it is important to be aware that psychological hardiness does not necessarily make an individual immune to the negative consequences of stress, as documented in the abovementioned empirical studies. Furthermore, it is also argued that one’s hardiness might change [31], even if it is argued that psychological hardiness is relatively stable across situations and over time [42]. Research has, for instance, shown that it is possible to develop and improve psychological hardiness [43], and that social support is a factor that strengthens hardiness [35]. This may indicate that psychological hardiness might also be impaired or weakened, especially so when facing ongoing long-term social stress. Because bullying is seen as a stressor that threatens and frustrates basic psychological needs [44], and accumulated long-term exposure to workplace bullying may be a serious, even traumatic, event, it is reasonable to expect a reversed relationship between the two. 

Cognitive dissonance might offer a theoretical explanation of how bullying may change individual dispositions, such as hardiness. A person of hardy disposition will probably expect to handle exposure to bullying behavior in the same way as she/he normally handles challenges—they become involved, see the challenge, get control, and cope with it [31,33]. However, since bullying is a strong stressor that most people do not cope with, even a person with a hardy disposition will most likely experience a discrepancy between the expectation (coping) and what really happens. These contradicting experiences may result in cognitive dissonance—an experience that normally triggers a discomfort that motivates the person to reduce the perceived dissonance [45]. Theoretically, this might happen by altering one’s cognition, changing one’s behavior, or adding new cognitions. Changing one’s behavior in order to avoid the perpetrators by, for example, seeking a new position might be one alternative solution for reestablishing cognitive consonance. However, it is also likely that helplessness or hopelessness will be established as a new cognition [28]. As a result, we might expect a deterioration of psychological hardiness in targets of accumulated long-term exposure to bullying behaviors at work.

### Aim of the Study

The causal associations between individual characteristics and bullying may be multifold [9]. Hence, by employing longitudinal data, we examine whether accumulated and long-term exposure to bullying behaviors at work will deplete an individual’s inner resources and reduce their psychological hardiness, as also hypothesized by Zapf and Einarsen [19]. However, we might also theoretically expect a forward causation, whereby people low in hardiness may face more bullying over time, because they either appear to be easy targets or are unable to cope with and stop the unwanted treatment. A vicious circle of events might also develop between bullying and changes in personal hardiness, in that people low in hardiness may experience more bullying, which again might reduce their levels of hardiness, making them even more prone to being targeted.

For both theoretical and applied reasons, this paper will investigate the relationship between exposure to bullying behavior in the workplace, and individual disposition in the form of psychological hardiness, focusing on *the reverse causal mechanism* perspective, yet also testing for a forward causation. Knowledge on how hardiness and exposure to workplace bullying are related might be of importance to both managers and occupational health personnel as it may inform both about preventive measures as well as interventions in actual cases. In addition, it might be relevant for treating the aftermaths of bullying experiences. It may also be important knowledge to those targeted, as it might help in normalizing their feelings and reactions. 

**Figure 1:**
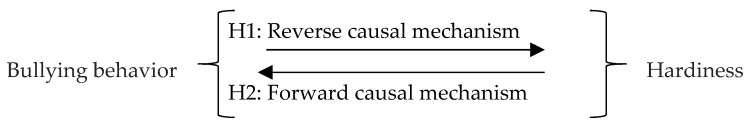
This figure shows how bullying hypothetically can be related to the personality disposition hardiness.

Therefore, based on the theoretical framework presented above, the following hypotheses were tested (see also Figure 1):

**Hypothesis** **1** **(H1).** *Accumulated long-term exposure to workplace bullying behaviors is related to subsequent reduced psychological hardiness, controlled for psychological hardiness at baseline.*

**Hypothesis** **2** **(H2).** *Hardiness predicts subsequent exposure to workplace bullying behaviors, controlled for exposure to workplace bullying at baseline.*

## 2. Materials and Methods

This study is based on longitudinal data from a nationwide cohort study of Norwegian nurses, Survey of Shift work, Sleep and Health (SUSSH), including five annual waves of data collection. The Regional Committee for Medical and Health Research Ethics in Western Norway approved the study (No.088.88). A total of 2059 (response rate = 38.1%) nurses, randomly selected from the Norwegian Nurses Organization’s membership roll of 2008, participated in the study. In 2009, the cohort was expanded with 905 (response rate = 33%) newly graduated nurses. To be included, one had to work at least 50% of a full-time position at baseline. Nurses who participated at time 1 (T1) in 2008/09 were invited to participate in annual follow-ups. The present study is based on data from the first five waves (T1–T5), where instruments measuring exposure to bullying behaviors and hardiness were included. From T3–T5, data from the two cohorts were collected at the same time and with a high response rate in all waves (69.4% to 78.5%). Questionnaires were distributed to the participants’ home addresses through the postal service, along with a prepaid return envelope. Two reminders were sent to those who did not respond. Participants who responded were placed into a lottery, where the prizes were 25 gift cards each worth 500 NOK (about 50 euro). The same procedure was used in all waves. 

### 2.1. Instruments

The questionnaires included information regarding age and sex (T1), exposure to bullying behaviors at the workplace (T1–T4), and hardiness (T1 and T5). 

Exposure to bullying behaviors at the workplace was measured with the Short Negative Acts Questionnaire [S-NAQ; 46]. The scale consists of nine items, measuring exposure to bullying behaviors that were experienced over the last six months. The items refer to both personal and work- related forms of bullying (e.g., “there has been gossip or rumors spread about you” and “necessary information was withheld that impeded your ability to do your job”). The response categories ranged from 1 (never) to 5 (daily). The instrument does not use the term bullying or harassment; hence, the respondent does not have to categorize him or herself as “bullied” or “not bullied”. A mean score was calculated if the participants had answered on at least seven out of nine items. S-NAQ has high reliability and validity and is recommended for assessing exposure to bullying behaviors at the workplace [46]. Cronbach’s alpha for exposure to bullying behaviors was 0.71, 0.77, 0.80, and 0.80 at T1–T4, respectively.

Psychological hardiness was measured with the revised Norwegian adaptation of the Dispositional Resilience Scale 15 [DRS-15; 47]. The scale consists of 15 items, measuring the individuals’ hardiness (e.g., “how things go with me in life depends on my own actions” and “I enjoy the challenge when I have to do more than one thing at a time”). The response categories ranged from 0 (completely disagree) to 3 (completely true). A mean score was calculated if the participants had answered on at least 12 out of 15 items. The Norwegian version of DRS-15 has been investigated for reliability and validity and it is recommended for assessing psychological hardiness [47]. Cronbach’s alpha for psychological hardiness was 0.74 and 0.79 at T1 and T5, respectively. 

### 2.2. Statistics

The data were analyzed using IBM’s SPSS Statistical package (IBM Corp, version 25.0, Armonk, NY, USA). Pearson’s product-moment correlations were calculated to study the association between study variables and Cronbach’s α coefficient was used to measure the internal consistency of the scales. Preliminary analyses were conducted to ensure that there was no violation of the assumptions of normality, multicollinearity, linearity, and homoscedasticity for regression analyses. A *p*-value < 0.05 was regarded as being statistically significant. A hierarchical multiple linear regression model was applied to investigate the effect of long-term accumulated exposure to bullying behaviors on psychological hardiness. In step 1, age, sex (male = 1, female = 2), and psychological hardiness at baseline (DRS-15, T1) were entered as the control variables. In step 2, the accumulated exposure to bullying behaviors (sum of S-NAQ from T1 to T4, if the participants had completed all four measure points) was entered. The dependent variable was psychological hardiness (DRS-15), as measured at T5. A second model was applied to investigate the forward causation hypothesis, with hardiness as a predictor of exposure to bullying behavior at the workplace. In step 1, age, sex and exposure to bullying behaviors at baseline (S-NAQ, T1) were entered. In step 2, psychological hardiness (DRS-15, T1) was entered. The dependent variable in the equation was exposure to bullying behaviors (sum of S-NAQ from T2 to T4). The difference between the focal standardized regression coefficients in the two models was evaluated using a Z-test [48]. Only participants who completed the S-NAQ and the SRS-15 scales in all five waves were included in the analyses.

## 3. Results

A total of 939 nurses completed the S-NAQ and the SRS-15 scales in all waves. At T1, the study included 91% women and 9% men, with a mean age of 34 years, ranging from 21 to 63 years. The mean score of psychological hardiness and exposure to bullying behaviors for the total sample was stable during the study period (Table 1). The analyses showed a weak negative correlation between exposure to bullying behaviors at the workplace and psychological hardiness at all-time points. 

The regression analysis revealed that accumulated long-term exposure to bullying behaviors predicted changes in psychological hardiness (F_4,938_ = 118.80, *p* < 0.001) (Table 2). Thus, H_1_ was supported. The accumulated long-term exposure to bullying behaviors explained 2.3% of the change in psychological hardiness (*β* = −0.16, t= −5.70, *p* < 0.001). 

The results from the second model testing the forward causation hypothesis revealed that low hardiness at baseline (T1) predicted a subsequent higher exposure to bullying behaviors (T2–T4), (F_4,937_ = 137.95, *p* < 0.001) (Table 3). Thus, H_2_ was supported. Hardiness at T1 explained an additional 0.3% of the change in exposure to bullying behaviors (*β* = −0.05, *t =* −2.04 *p* < 0.05).

A comparison of the focal standardized regression coefficients from the two models above showed that exposure to bullying behaviors was a significantly stronger predictor for changes in hardiness than baseline hardiness was for changes in exposure to bullying behaviors (Z = 2.66, *p* < 0.01).

## 4. Discussion

The present study shows that accumulated exposure to bullying behaviors over a four-year period had a negative impact on psychological hardiness. Hence, nurses that are subjected to long-term exposure to bullying behaviors are likely to, over time, display a deterioration of psychological hardiness that might decrease their ability to withstand and cope with stress. Yet, our results suggest that also the opposite, in fact, holds true. Indeed, less hardy individuals reported a somewhat higher subsequent exposure to bullying behaviors at T5. This supports the contention that the temporal associations between personality and bullying can be complex and multiple [9]. Still, our analyses indicate that the reversed causal mechanism is stronger than the forward causal mechanism, meaning that bullying is a stronger predictor of changes in hardiness, in comparison to the ability that hardiness has in predicting subsequent bullying exposure. Early works in the field of workplace bullying support this finding. Based on case studies, Leymann [13] claimed that distinct personality features, such as neuroticism, introversion, conscientiousness, and submissiveness, should be considered to be the result of enduring bullying, rather than the initial cause. Some new evidence indicating changes in personality due to bullying also exists from more recent empirical studies [15,16,49], supporting the *reverse causal mechanism* perspective. In a prospective study, it was observed that victimization from bullying at baseline was followed by changes in agreeableness, conscientiousness and openness [50]. Changes in agreeableness following bullying were supported in a prospective study [49], showing that enduring bullying led to a reduction in the victim’s capability to display empathy and trust [51]. 

Conversely, the analyses of the present study also indicated that hardiness was inversely related to subsequent exposure to bullying behaviors, a finding that was supported by evidence indicating that personality traits predict subsequent exposure to bullying [50]. Less hardy individuals reported a higher incidence of bullying. This might be explained by their actual experience of more incidence of bullying, their diminished ability to defend against negative actions or by the fact that less hardy individuals are more susceptible to recognizing negative acts or interpret actions as more hostile than their counterparts. The current study design prevents any specific conclusion regarding underlying mechanisms, beyond lending some support to the forward causal mechanism. Our hypotheses were not mutually exclusive and point to the existence of a possible vicious circle. Yet, our findings indicate that bullying behaviors impact hardiness more than the other way around. 

As far as the authors can ascertain, hardiness has until now neither been considered as a predictor of subsequent bullying nor as an outcome of bullying, but merely as a protective [29] and moderating mechanism in the face of stressful events [37]. Theoretically, an individual with resilient qualities, such as hardiness, should be protected when exposed to bullying and there is evidence indicating that psychological hardiness protects against critical incidents similar to being bullied [29]. However, this might not be the case when dealing with accumulated exposure to bullying behavior, as revealed by the present study. Thus, persistent exposure to bullying behaviors might be an extreme stressor, causing the victims severe distress and impacting their perceptions, coping mechanisms, and their capacity to develop self-efficacy, even changing their personality. 

This finding contrasts with general stress theory, which claims that hardy properties, like having a positive outcome expectancy, act as protective factors when one encounters stressful life events [33]. Normally, it is expected that hardy individuals perceive a stressor, or a challenging situation, as a potential opportunity for growth [32]. However, the findings are in accordance with other studies showing that bullying is such a severe stressor that none known individual protective factors exist [9].

According to CATS, positive coping expectations will affect the outcome of a challenging situation [33]. If there is no amelioration of a bullying situation, this might lead to exposure to the accumulated perception of bullying behavior where the bullying might even escalate with time [52]. Given this, a target with positive coping expectation may experience a strong cognitive dissonance, which, according to the theory of cognitive dissonance [45], will be very desirous of recovering cognitive harmony. When there are no accomplished changes and improvements in the objective situation, subjective changes in the victim’s coping expectations are one intrinsic solution that may re-establish cognitive equilibrium. Hence, reduced psychological hardiness might be a consequence of exposure to accumulated bullying behavior. This is supported by a recent publication proposing that the interaction between the experience of bullying and the coping strategies adopted defines the personal outcome [41]. 

From a wider perspective, numerous cross-sectional and prospective studies show that individual dispositions, which are traditionally associated with protective and coping-enhancing factors, are less beneficial than might be expected when targets are exposed to high levels of bullying behaviors over time [9]. It is suggested that this is due to loss of control [40], which simultaneously impedes the victim’s basic need for autonomy. This line of argument is supported by findings showing that exposure to bullying behavior threatens the satisfaction of an individual’s basic psychological needs [17] and frustrates their need satisfaction [44]. Thus, individuals chronically exposed to thwarting environments, such as long-term exposure to bullying behaviors, are expected to develop fewer resources for growth, which again can foster feelings of personal oppression and inadequacy [44]. Evidence on individual mechanisms for coping with personal distress regards bullying as a situation beyond the target’s immediate control [19], and in the absence of any improvements in the external situation an unpleasant psychological state of tension will manifest. The dissonance is assumed to be higher among those with higher initial levels of hardiness/coping resources. For personality change to occur, it is generally emphasized that the stressor is present over time [53]. Bullying represents such a stressor [19], and it can be differentiated from other stressors as being both episodic and chronic. Stress concepts are generally concerned with the importance of resources: exposure to bullying is known to be a predicament occurring when an individual has low external and internal resources. The chronic progression of bullying considerably restricts and narrows the afflicted person’s spectrum of vitality [24]. Furthermore, being discovered by studying bullying cases, victims’ previous attitudes and behaviors corresponding to “ideal” personality structures failed to adequately protect the victim in the actual situation, hence overriding hardy qualities [24]. 

The present study has some noteworthy strengths and limitations. The study employs a large sample followed over five years, using well-established instruments. The response rate at baseline was rather low, even though all follow-up waves had response rates above 69.4%, indicating that participants who first agreed to participate were committed. Second, the present study focused on bullying in a profession dominated by females and, in particular, nurses. This might limit generalization to other work domains, and replications should thus be carried out in other work settings. Furthermore, as the short Negative Acts questionnaire was kept in its validated format [46], assessing exposure to bullying behaviors over the last six months, any exposure to bullying behaviors experienced in the six months directly following the completion of the annual questionnaire is not taken into account in the present study. Consequently, the strength of the relationship between exposure to bullying behaviors and subsequent changes in hardiness might be underestimated. Fourth, the analyses did not distinguish between the effect of low versus high exposure to bullying behavior on a given time point. The respondents experiencing relatively low rates of bullying behavior in all four years, and respondents with short-term, but high exposure to bullying behavior may obtain the same average score. Yet, the results indicate that accumulated long-term exposure is related to lowered hardiness longitudinally. Finally, a 2.3% change in explained variance with a standardized beta coefficient of −0.16 might not seem much at first glance. However, personality traits like hardiness are supposed to be rather stable, thus large changes could not be expected. Furthermore, exposure to bullying behavior is a stressor of relatively low frequency.

## 5. Conclusions

The present study showed that accumulated exposure to bullying behaviors predicted reduced hardiness in nurses, thus supporting the reversed causal mechanism. A smaller effect was found regarding the opposite direction, supporting the forward causation mechanism. Taken together, this supports the notion that the relationship between bullying and personality might be multifold and, to some extent, constitute a vicious circle. Beyond earlier evidence showing that bullying influences health, managers as well as occupational health personnel should also be aware that exposure of bullying behaviors at the workplace seemingly impairs psychological hardiness. Thus, exposure to bullying behaviors at work might not only impair the targeted employees’ health and well-being in itself, but might also reduce employees’ coping resources, leaving them more vulnerable to future workplace bullying or other challenging and stressful circumstances. The findings illustrate the importance of preventing bullying, and actively intervening when bullying does occur in order to minimize the subsequent consequences.

## Figures and Tables

**Table 1:** Means, standard deviation (SD), and correlation between age, psychological hardiness, and exposure to bullying behaviors at the workplace.

Variable	Mean (SD)	1	2	3	4	5	6	7	8
1. Age (T1)	33.76 (8.44)	-							
2. Hardiness (T1)	2.10 (0.29)	0.06	-						
3. Hardiness (T5)	2.11 (0.35)	−0.02	0.56 **	-					
4. Bullying (T1)	1.18 (0.26)	0.01	−0.18 **	−0.19 **	-				
5. Bullying (T2)	1.18 (0.27)	0.00	−0.15 **	−0.021 **	0.54 **	-			
6. Bullying (T3)	1.18 (0.29)	0.01	−0.13 **	−0.22 **	0.51 **	0.53 **	-		
7. Bullying (T4)	1.18 (0.30)	−0.01	−0.13 **	−0.20 **	0.49 **	0.50 **	0.64 **	-	
8. Bullying (T1–T4)	1.18 (0.23)	0.00	−0.18 **	−0.25 **	0.77 **	0.79**	0.84 **	0.82 **	-
9. Bullying (T2–T4)	1.18 (0.24)	0.00	−0.16 **	−0.25 **	0.61 **	0.80 **	0.87**	0.86**	0.97 **

** *p* < 0.01.

**Table 2:** Hierarchical multiple regression analysis of exposure to bullying behaviors from T1 to T4 predicting psychological hardiness at T5, controlled for age, sex, and psychological hardiness measured at T1. Unstandardized beta coefficient (B) with standard error (SE B), and the standardized beta coefficient (*β)* are reported.

Variable	Step 1			Step 2
	B (SE B)	*β*	B (SE B)	*β*
Age (T1)	−0.00 (0.00)	−0.04	−0.00 (0.00)	−0.04
Sex (T1)	0.07 (0.03)	0.06 *	0.06 (0.03)	0.05
Hardiness (T1)	0.66 (0.03)	0.55 ***	0.62 (0.03)	0.53 ***
Bullying (T1–T4)			−0.24 (0.04)	−0.16 ***
Δ*R^2^*		0.314		0.023
*F* for Δ*R*^2^		142.81 ***		32.51 ***

* *p* < 0.05, *** *p* < 0.001.

**Table 3:** Hierarchical multiple regression analysis of exposure to workplace bullying (sum of bullying from T2 to T4) predicted by hardiness at T1, controlled for age, sex, and workplace bullying measured at T1. Unstandardized beta coefficient (B) with standard error (SE B), and the standardized beta coefficient (*β)* are reported.

Variable	Step 1			Step 2
	B (SE B)	*β*	B (SE B)	*β*
Age (T1)	0.00 (0.00)	−0.00	0.00 (0.00)	−0.00
Sex (T1)	0.00 (0.02)	0.01	0.01 (0.02)	0.01
Bullying (T1)	0.56 (0.02)	0.61 ***	0.55 (0.02)	0.60 ***
Hardiness (T1)			−0.04 (0.02)	−0.05 *
Δ*R^2^*		0.374		0.003
*F* Δ*R*^2^		223.87 ***		4.90 *

* *p* < 0.05, *** *p* < 0.001.
